# TB patients learning about second hand smoke (TBLASS): a pilot individual randomised controlled trial

**DOI:** 10.1186/2193-1801-2-556

**Published:** 2013-10-24

**Authors:** Nauman Safdar, Raana Zahid, Sarwat Shah, Ian Cameron, Razia Fatima, Huma Qureshi, Kamran Siddiqi

**Affiliations:** Social and Health Inequalities Network, 862, St 13-C, E-11/4, Islamabad, 44000 Pakistan; Department of Health Sciences, University of York, York, YO10 5DD UK; Leeds City Council, Leeds, UK; National TB Control Programme, Block E & F, EPI Building, Near National Institute of Health (NIH) (Prime Minister’s National Health Complex), Park Road, Islamabad, Pakistan; Pakistan Medical and Research Council, Shahrah-e-Jamhuriat, G-5/2, Islamabad, Pakistan; Department of Health Sciences, Hull York Medical School University of York, York, YO10 5DD UK

**Keywords:** Tobacco, Tuberculosis, Second hand smoke, Smoke free home

## Abstract

**Background:**

Living with a smoker is a key determinant of exposure to Second Hand Smoke (SHS) and its exposure mainly occurs at home. Exposure to SHS from tobacco in the household predisposes to the development of tuberculosis (TB) and outcome of the disease gets worse. We aim to develop and evaluate a behavioural intervention 'Smoke Free Homes’ (SFH) for TB patients that encourages them to negotiate a smoke free environment within their homes.

**Methods and design:**

The pilot individual randomised controlled trial of SFH will inform the design of a future definitive trial. We will first develop SFH intervention using taxonomy of behaviour change techniques aimed at encouraging families of non-smoking TB patients to implement smoking restrictions at home following a logic model of the intervention. This will be followed by conducting a pilot randomised controlled trial of intervention within the context of routine TB control programme. The eligible non-smoking TB patients will be randomised and allocated to one of the two trial arms consisting of “individual based care” and “individual based care” plus “supplementary support”. We aim to recruit 150 newly registered pulmonary TB patients from two selected TB centres with 75 cases in each arm. The *Primary outcome* measure will be SFH of non-smoker TB patient by validating through 'Urine Cotinine’ test. We will also determine qualitatively the barriers and key drivers to the creation of smoke free homes followed by developing a definitive trial.

**Discussion:**

The male to female distribution of TB cases in Pakistan is almost equal whereas, tobacco use among males is much high as compared to females in Pakistan. This reflects a strong possibility that women health can be affected by men behaviour. Appropriate storage, restricted access and disposal arrangements for participant’s personal details will be implemented. All ethical issues will be addressed. There will be no extra burden, financial or otherwise, on the participants. They will not receive any financial incentive to participate in the study.

**Trial registration:**

Trial Registration Number: ISRCTN83630841

## Background

### Second hand smoke

Second-hand smoke (SHS) is a combination of 'side stream’ smoke from the burning tip of a cigarette and 'mainstream’ smoke exhaled by the smoker (Fielding and Phenow 1988). Toxic chemicals from SHS cling to rugs, curtains, clothes, food, furniture and other materials. These toxins remain in the environment despite open windows, fans or air filters; and can recycle back into the air through the filters and coat the surfaces of rooms, materials and smoker's belongings (World Health Organization 2009). Second-hand smoke kills 600000 people each year and its exposure contributes to an increased risk of cardiovascular diseases, lung cancer, asthma and other respiratory diseases both in adults and in children (World Health Organization 2009; Reddy and Gupta 2004). Living with a smoker is a key determinant of exposure to SHS and it occurs mainly in homes. The General Adult Tobacco Survey in India shows that 52% of the adults (rural-58%, urban-39%) are exposed to SHS at home (Global Adult Tobacco Survey (GATS) India 2010).

### Tuberculosis, smoking and second hand smoke

Smoking was independently associated with significantly increased risks of latent TB infection (LTBI). In certain populations, a greater risk of latent TB corresponded with increased smoking exposure is also observed (Horne et al. 2012). Exposure to SHS from tobacco in the household predisposes to the development of tuberculosis (TB) (Leong 2010). In addition, outcomes in TB patients gets worsen and risk of TB transmission increases if there is a continued exposure to SHS from tobacco. There is evidence showing significant association between SHS with drug resistant TB, severity of the disease, patient delay smear conversion and death during or after treatment. A systematic review and meta-analysis of observational studies reporting on relation of TB and tobacco revealed substantial evidence that tobacco smoking is positively associated with TB and SHS increases the risk of TB disease (Lin et al. 2007). Children are at high risk of acquiring TB infection that lives with adults who have had TB and are also smokers (Patra et al. 2012; Singh et al. 2005; Alcaide et al. 1996). Moreover, children who have had a recent TB infection are also five times more likely to develop active TB disease if exposed to SHS compared to children living in a smoke free environment (Alcaide et al. 1996).

### Smoking restrictions at home

It is crucial for better health of a family that their home should be free from tobacco smoke, as around 90% of the exposure to SHS takes place at home. This calls for restrictions for smokers to smoke inside the home which could be only achieved by rigorous work by identifying the smoker and convincing him/her not to smoke inside the home thus making a Smoke Free Home (SFH). Factors such as, younger age, being married, dislike for second hand smoke, and personal smoking behaviours such as smoking every day, on occasions, etc were found associated with having home and workplace smoking bans (Heck et al. 2010). In few cases, parents with younger children in home appear to have higher uptake of smoking bans than parents of older children or adolescents (Merom & Rissel 2001; Martinez-Donate et al. 2009). A qualitative study conducted in Scotland revealed that; the enablers to creating smoke free environment within the home include increasing awareness of the risks of SHS particularly in relation to children, desire to be seen as behaving in morally and socially acceptable ways, social norms about the unacceptability of smoking in the home among family and friends including pressure from children. Whereas, the barriers include limited understanding of and resistance to messages about the health risks of SHS, the need to smoke and smoker identity, the home perceived as a private space protected from public controls and sanctions, social norms among family and friends about the acceptability of smoking in the home (Philips et al. 2007). Awareness of the risks of SHS, despite ambivalence about health messages and the fluidity of smoking restrictions provides clear opportunities for public health initiatives to support people attain smoke-free homes.

#### Research problem and justification

Pakistan is low income country; and is known to have a 5^th^ highest TB burden and 9^th^ highest tobacco use among men in the world (World Health Organization 2012; Third WHO Report on the Global Tobacco Epidemic 2011). According to the World Health Survey, Pakistan (2002-2003), in adults 18 years and above prevalence of tobacco smoking was 19.1%, with males being 32.4% and females 5.7% respectively. In a survey conducted in one of the districts of Pakistan in 2008; 16.5% of the study population (33% men and 4.7% women) were found using tobacco on daily basis. The prevalence of exposure to SHS was found to be 56% (35% were exposed to SHS on daily basis and the remaining were exposed few times a week) (Alam et al. 2008). A survey of women on tobacco use and exposure to SHS during pregnancy showed that in Pakistan the SHS exposure in women was high. 91.6% homes allowed smoking indoors, 49.9% were frequently/always exposed to tobacco smoke indoors and 51.4% reported their young children being frequently/always exposed to tobacco smoke indoors (Bloch et al. 2008).

The government of Pakistan being the signatory of Framework Convention for Tobacco Control (FCTC) has already taken a number of important steps for effective tobacco control in the country including restriction on advertisement, health warning on cigarette packs, ban on sales to minors, mass media awareness, and legislations. According to current policy smoking is banned in health and educational facilities, offices and on public transportation. However, there are no clear guidelines and evidence is lacking on how to ensure a smoke free environment inside home for non smokers. There is currently no evidence on interventions, which could help to reduce exposure to SHS for non-smoker TB patients and help improve their TB outcomes by making their homes smoke free. This embarks on a need for innovative solutions within the context of TB care delivery which could provide evidence for creating SFH for TB patients which will be likely the main contribution of our proposed study. Similar interventions are being testing in educational settings (Siddiqi et al. 2010), but not done in the health care setting in risk groups such as TB patients. This is for the first time a pilot Randomized Control Trial (RCT) is going to be implemented in TB patients in making their home smoke free. Furthermore, such approaches have been highlighted in the declaration on Non Communicable Disease (NCDs) in United Nations summit held in September, 2011 (UN Sumit 2011) and also in World Health Organization 'MPOWER package’ (The MPOWER package 2008) and The Union 'Smoking cessation and smoke free environment for TB patients’ (Bissell et al. 2010).

#### Research questions

This pilot trial has been designed to answer the following research questions;

1. What behaviour change techniques are relevant and appropriate to be used in the development of 'Smoke Free Homes’ intervention?
2. What is the feasibility and fidelity of implementing 'Smoke Free Homes’ intervention in TB patients?
3. What is the proportion of non-smoker TB patients among all registered TB cases and what is the smoking behaviour of residents in their homes?
4. What is the rate and reasons of refusal to participate in the research by the non-smokers with smoker in home?
5. What are the recruitment and attrition rates for participants?
6. What is the likely effect size in relation to the primary outcome measure i.e. reduction in the exposure to second-hand smoke validated by a urinary cotinine test after 2 months of TB treatment?
7. What is the feasibility and acceptability of measuring the primary outcome?
8. What are the barriers of change reported by the TB patient during follow-up?
9. What is the feasibility of measuring TB treatment outcomes among trial participants?

#### General objectives

The purpose is to improve outcomes among those TB patients who are non-smokers themselves but are exposed to SHS and reduce the risk of TB transmission to other members of the household by encouraging families to make their homes smoke free.

#### Specific objectives

We aim to develop and evaluate a behavioural intervention 'Smoke Free Homes’ for TB patients. SFH for TB patients’ is a behavioural intervention that encourages TB patients to negotiate a smoke free environment within their homes. Our specific objectives are:

1. To develop a behaviour change intervention 'Smoke Free Homes’ aimed at encouraging families of newly registered non-smoking TB patients to implement smoking restrictions at home (Phase I).
2. To conduct a pilot randomised controlled trial of 'Smoke Free Homes’ intervention within the context of routine TB control programme (Phase II).
3. To determine the barriers and key drivers to the creation of SFH (Phase III).
4. To develop and submit a proposal on a definitive trial based on the pilot trial findings (Phase IV).

## Methodology

### Design

This is a pilot individual randomised controlled trial of SFH that will inform the design of a future definitive trial. The definitive trial will assess the effectiveness and cost effectiveness of SFH intervention in reducing SHS exposure for non-smoking TB patients compared to routine care. We will first develop SFH intervention using taxonomy of behaviour change techniques (Michie & Abraham 2008) in order to help TB patients in negotiating smoking restriction at home. The selected Behaviour Change Taxonomy (BCTs) will guide the development of the content of the intervention tools likely to be in the form of a flip chart and a leaflet. This will be designed to inform and support TB patient to negotiate smoking restrictions in their homes.

### Objective 1

To develop a behaviour change intervention 'Smoke Free Homes’ aimed at encouraging families of non-smoking TB patients to implement smoking restrictions at home. (Phase I).

#### Key activities

**Figure 1:**
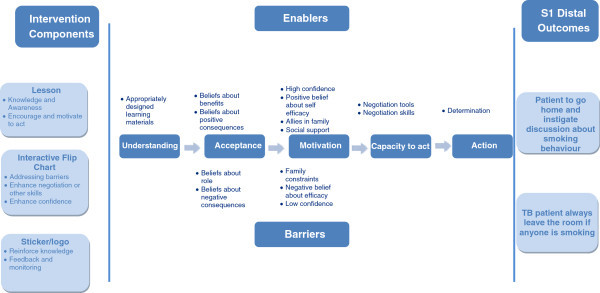
**Intervention stage 1: health professional to non-smoking TB patient (who are exposed to SHS at home).**

**Figure 2:**
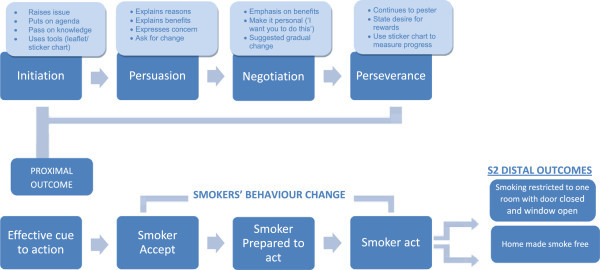
**Intervention stage 2: TB patients to smokers in the home.**

The process of intervention development will run over 6 months period that will also include developing and finalising trial protocol and research tool. The development will take place in a phased manner following a logic model of the intervention (Figures 1 and 2). The logic model provides a pathway and focus to develop educational materials that improve knowledge, attitude, motivation, build confidence and negotiation skills, and increase intention to act with further reinforcement at the follow-up. It will be a two-pronged intervention where stage-1 will focus on the interaction between 'care provider and TB patient’ and stage-2 on the interaction between 'non-smoking TB patient and smoker(s) at home’.

The process of development will include organising a Development Core Group (DCP). The members will include representatives from the national TB control programme and tobacco control cell, media and communications expert, psychologist and the research team. The group will review the draft materials and approve these for piloting.

#### The intervention and its components

The SFH intervention will be based on behaviour change taxonomy (BCTs). The intervention will follow the following steps to bring about a change in the behaviour of the smoker living with the non-smoking TB patient: a) providing information to the non-smoking TB patient; b) helping him/her to formulate a strategy to change behaviour of the smoker and help sustaining that change; and c) finally giving a clear call to action for behaviour change.

#### Outputs

1. A complete set of refined SFH intervention materials including an illustrative flip chart to inform and encourage non-smoking TB patient to negotiate smoking restrictions at home.
2. An illustrative leaflet for the TB patient that will help him/her to start a conversation on the topic of making the home smoke free.
3. The intervention package will also include supplementary support which includes mobile phone standardized text messages or letter (where mobile phone is not available) on a weekly basis, to be sent to smoker at home in the arm 2 of the trial.

Figures 1 and 2 presents the logic model which presents the flow of events and the expected outcomes.

### Objective 2

To conduct a pilot randomised controlled trial of 'Smoke Free Homes’ intervention within the context of routine TB control programme (Phase II).

#### Hypothesis

We hypothesis that the SFH educational materials with or without supplementary support (mobile phone text message 'SMS’ or letter to smoker(s) at home on weekly basis) will be effective in relative or absolute reduction of exposure to tobacco smoke at home in non-smoking TB patients living with smoker(s). The option of supplementary support of SMS or letter seems more powerful as this may persuade the smoker at home in implementing smoking restriction in home.

#### Setting

The trial protocol will be implemented and evaluated in two selected TB centres (within the premises of district hospitals) in the public sector which are designated TB diagnostic centres by the National TB Control Programme (NTP), and serve both rural and urban settings in two districts of the province of Punjab-Pakistan. These centres are routinely staffed by a doctor, designated TB facilitator and a laboratory technician. They are also equipped with a basic microbiology laboratory for sputum smear microscopy. These TB centres are busy out-patient settings and on an average deals with about 75-100 patients a day with limited human resource. A TB suspect is screened by the doctor (trained by NTP on TB case management protocols) supported by investigations and if diagnosed as TB case is registered by the TB facilitator (a paramedic trained on TB case recording and reporting) and offered six months of TB treatment. The follow-up of the case is also the responsibility of the doctor and the TB facilitator.

#### Study design and sample

**Figure 3:**
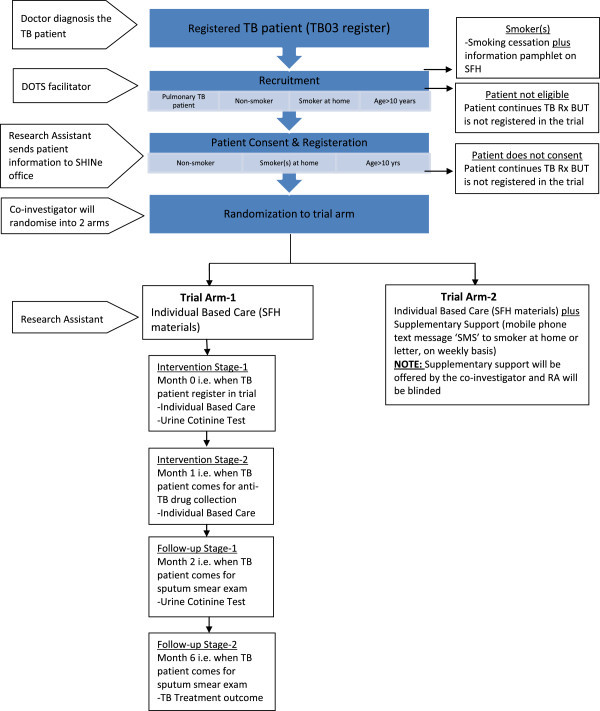
**Trial flow chart.**

The design of the trial is an individual randomised controlled trial (RCT). Figure 3 represents the overall flow of the events in the trial. To deliver 'Smoke Free Home’ intervention, the eligible non-smoking TB patients will be randomised and allocated to one of the two trial arms. **Arm 1** consists of “individual based care” i.e. SFH intervention materials, whereas **Arm 2** consists of “individual based care” plus “supplementary support” i.e. mobile phone text message or letter to smoker(s) at home with an assumption that it would persuade more the smoker to smoke outside the home. As it is a pilot RCT, we aim to recruit 150 newly registered pulmonary TB patients from two selected TB centres with 75 cases in each arm.

#### Inclusion criteria

- Should be a registered case of new pulmonary TB, either sputum smear positive or negative ( *new TB patients are defined as those who have no history of prior TB treatment or who received less than 1 month of anti-TB drugs, regardless of whether their smear or culture results are positive or not).*
- Should be a non-smoker.
- Have not taken part in a 'Smoke Free Homes’ activity before.
- Residing within the same district.
- Aged more than 10 years.
- Lives with at least one other person who smokes tobacco on a regular basis without any restrictions in the house.

#### Recruitment

The recruitments will start in November 2012 and will continue until we recruit the targeted number of participants. Each participant in the cohort will be followed-up for 6 months after the inclusion in trial. It will be led by the TB facilitator in the TB centre. The following process will be followed to recruit the participants. The TB facilitator will screen all newly registered pulmonary TB patients for inclusion in the trial. The TB facilitator will first confirm that the TB patient is a) case of new pulmonary TB by reviewing the TB register, b) non-smoker by prompting; do you smoke tobacco? such as cigarette, beeri, huqa, cigar, any other (once daily a cigarette/pack, huqa session(s) or any other form of tobacco), c) resident of the same district by reviewing the TB register, and d) aged more than 10 years by reviewing the TB register. All such patients who meet the above criteria and are eligible will be referred to research assistant (RA) for further trial procedures. The TB facilitator will continue offering TB care to all registered patient as per NTP protocol, irrespective of their trial status.

#### Trial registration and informed consent

This will be led by the RA based in the TB centre. The RA will record patient basic information, eligibility and consent. If the patient meets the eligibility criteria the RA will continue with informed consent process.

RA will seek written informed consent from all participants who full fill the eligibility criteria of the trial activities. The RA will use the 'Information Sheet’ to inform the eligible patients about the nature and purpose of the trial; potential risks and benefits of participation in the trial including consequences of participating and not participating in the trial. If the patient does not agree to participate the RA will not proceed with the registration process and will record the reasons for non participation on the specified place in the registration form. The patient will continue TB routine care management at the health facility. We are not offering any personal incentive to the participant for taking part in the study.

#### Patient randomisation

The research team will draw simple randomisation tables where the unit of randomisation will be individuals. The 150 cases will be randomised in to two arms with 75 cases in each arm. The record of randomisation codes and allocation will be maintained by the principal investigator. Once randomised, a participant cannot be withdrawn from the trial. The RA will remain blinded to the fact regarding which arm has been allocated to the patient and will deliver the individual based care to all patients registered in the trial. Once a patient will be registered the information will be send by the RA to the principal investigator who will allocate the arm and inform the co-investigator. The SMS or letter to the arm 2 registered patients will be send by the researcher away from the TB centre. The trial register will be kept in researcher office under lock and key and only the principal investigator will have access to it.

#### Withdrawal

There are possibilities that a patient may decide to discontinue his/her participation in the trial at any stage with or without information. In case, the TB patient discontinues participation in trial during the first month, he/she will be declared non-adherent. Non adherence is defined as any departure from the current approved version of the trial protocol, so that the intervention is not completed. The RA will attempt to retrieve all such patients. In case, the TB patient discontinues participation in trial after the first month, he/she will be declared as 'loss to follow up’. The RA will not attempt to retrieve such patients; however, he will record their TB outcomes at 6 months. The retrieval process will be followed by the TB facilitators as per the TB control protocols. In case, a participant withdraws consent to participate, no further data will be collected from them. However, previously collected data will be used in analyses.

#### Data collection

The data will be recorded on trial registration by the RA. The co-investigator will be responsible for maintaining the quality of data collected. A code plan will be prepared to help data entry using SPSS software and the same will be used for analysis.

#### Outcome assessments

All outcomes will be measured after the intervention in each of the study’s arms. The *Primary outcome* will be SFH of non-smoker TB patient by validating through 'Urine Cotinine’ test. During the trial process two urine samples will be collected for cotinine testing at time of registration '0 month’ and at completion of '2 month’ of treatment. The first test will establish the base line where as the test at 2 month will be used to validate the effect of intervention. The urine cotinine testing will be done by the laboratory technician in the TB clinic who will receive relevant training on the conduct of the test and its interpretation. The RA will be kept blinded to the results of urine cotinine and the results will be collected by the researchers directly from the laboratory.

The *Secondary outcomes* will be mainly patient cure rates. All type of patient treatment outcome will be documented including; cured, completed, default, died, failure and transfer out.

#### Data monitoring

Data will be monitored for quality and completeness by the principal and co-investigator using verification, validation and checking processes. Missing data will be pursued until study end unless they are data obtained by participant contact and this would cause distress.

#### Data analysis

Summaries of the baseline characteristics of participants will be presented by trial arm, and recruitment and attrition rates will be reported. Although it is a pilot trial and the ample size has not been powered to estimate the likely effect size of the primary outcome, a comparison between the two intervention arms will be undertaken to determine the difference in the outcome between two arms adjusting for health centre. Differences of the secondary outcome between the two arms will also be reported. Analyses will be conducted under the principle of intention to treat, with individual participant as the primary unit of analysis.

### Objective 3

To determine the barriers and key drivers to the creation of smoke free homes. (Phase III).

#### Key activities

We will require an in-depth understanding of the way the intervention has worked within the household setting. This implies the knowledge to be gained from the patients on actual drivers and barriers to effectively impose smoking restrictions in the home. We would be interested in understanding in which ways the patient felt supported in their efforts to keep their home free of smoke, which aspect of the SFH materials were most helpful, which factors motivated the smokers at home to change their behaviour, and which were their main constraints. Since this phase will be post intervention, it would be preferable to use semi structured interviews with key informants to gain this understanding.

#### Participants

The participants will be those from which we have received follow-up urine cotinine result i.e. at the end of 2 months of treatment. There will be 12 participants, six from each arm and representing both hospitals and will be grouped as;

- Patients who were successful in imposing smoking restriction in their homes.
- Patients who were unsuccessful in imposing smoking restriction in their homes.
- Patients who were partially successful in imposing smoking restriction in their homes.

#### Data collection

We will use an interview checklist and all communications will be tape-recorded and transcribed.

#### Data analysis

The data will be categorized for thematic analysis.

#### Outputs

The understanding gained from this activity will be used to further refine the intervention of SFH individual based care and supplementary support to take account of barriers and enablers to administer smoking restrictions in homes. Moreover, the findings will be also helpful in designing the definitive trial.

### Objective 4

To develop and submit a proposal on a definitive trial based on the pilot trial findings. (Phase IV).

#### Key activities

The findings of the trial will be used to formulate and submit a definitive trial. The definitive trial will have a statistically valid sample size considering all the issue such as recruitment and attrition rate, etc based on pilot trial. The definitive trail will be developed in collaboration with University of York, United Kingdom and National TB control and Tobacco control programme Pakistan.

## Discussion

### User participation

We will not make any specific efforts to increase user participation. However, the RA will keep a close liaison with the TB facilitator and will be trained to communicate the health benefits of the intervention to the TB patients while requesting for consent.

### Gender and status considerations

The gender and status are key determinants of the proposed change in smoking behaviour in home. The male to female distribution of TB cases in Pakistan is almost equal whereas, tobacco use among males is much high as compared to females in Pakistan. This reflects a strong possibility that women health can be affected by men behaviour. The gender and relationship/status of the TB patient and that of the smoker at home, may create conflicting scenarios within the household setting. We will conduct interviews of both males and females to capture gender perspectives and status implications in administering smoking restrictions in the home.

### Confidentiality and data protection issues

All the patient data files, paper and soft copy, will be kept confidential. The patient name will not be entered, instead a unique identification number will be used to identify the patients. In this research it will be the patient TB registration number which is standardized by the NTP. During the data entry patient identification number will be used which can only be tracked backed if required. All the information on paper and computer will be held securely with the principal investigator in Islamabad office. Appropriate storage, restricted access and disposal arrangements for participant’s personal details will be implemented.

### Ethical considerations

The research questions address a priority need for non smoking TB patients in Pakistan as they are commonly exposed to second hand smoke. We understand that children’s (10-18 years) participation in such studies raises issues around incompetence, vulnerability and powerlessness. We will ensure that our approach to children is participatory and as non-intrusive and accommodating as possible. There may be possible issues with smoker in the household receiving letters and SMS messaging which could provoke domestic disturbance. RA will check with patient as part of consent that if the team communicates with the smoker will it cause any issues. Also if patients want to keep his or her illness confidential in which case it would not be mentioned in the message. No participating TB patient will be deprived of any service that she or he would ordinarily receive at the TB centre. There will be no extra burden, financial or otherwise, on the participants. The TB patient and their families will not incur any cost for participating in this study. They will not receive any financial incentive to participate in the study. The messages in the 'Smoke Free Homes’ intervention is not harmful and will be in Urdu language. All data collected will be confidential. The TB centre management will be provided complete information about the study.

#### Ethical clearance

The trial has received ethical clearance from the 'National Bioethics Committee’ Pakistan.

#### Trial status

The patient’s recruitment was started in November 2012, and 150 patients recruitment was completed in end May 2013. Each recruited patient has to be followed-up for 6 months after the inclusion in trial. It is expected that the last patient registered in May, 2013 will have 6 month follow-up completed by Nov-Dec 2013 after which all data will be retrieved from the field sites and data entry and cleaning will start.

## Results and discussion

This is a protocol for a pilot individual randomised controlled trial of 'Smoke Free Homes’ intervention and therefore, its findings will inform a future definitive trial. The potential impact of this research is likely to be high as it addresses a key public health priority, uses an innovative approach, and engages with TB patients and their families that are generally not engaged in research and not been done in Pakistan. We will publish papers relating to this trial that will include mainly the trial protocol, findings on the feasibility and fidelity of intervention and a qualitative study. We will produce a short summary of the results that can be distributed to all trial participants, including TB facilitators, doctors and the management of TB centre. The University of York, National TB control programme and Tobacco cell will be a part of the dissemination process. We will also disseminate the research findings in national and international scientific conferences and policy meetings.

## Abbreviations

SHS: Second hand smoke
TB: Tuberculosis
SFH: Smoke free homes
NHS: National health services
LTBI: Latent TB infection
FCTC: Framework convention for tobacco control
RCT: Randomized control trial
NCDs: Non communicable disease
BCTs: Behaviour change taxonomy
DCP: Development core group
NTP: National TB control programme
RCT: Randomised controlled trial
RA: Research assistant.
